# Subxiphoid Incisional Hernia Following Cardiac Procedures: A Narrative Review

**DOI:** 10.3389/jaws.2025.15219

**Published:** 2026-01-08

**Authors:** Elissavet Symeonidou, Ioannis Gkoutziotis, Sotirios Dinas, Albion Totsi, Efthymia Liaretidou, Dimitrios Damaskos

**Affiliations:** 1 School of Medicine, Aristotle University of Thessaloniki, Thessaloniki, Greece; 2 Department of General Surgery, Princess Royal University Hospital, King’s College Hospital, London, United Kingdom; 3 5th Department of Surgery, Ippokratio General Hospital, Aristotle University of Thessaloniki, Thessaloniki, Greece; 4 Department of Surgery, Papageorgiou General Hospital, Thessaloniki, Greece; 5 1st Department of Cardiac Surgery, Onassis Cardiac Surgery Center, Athens, Greece; 6 Department of General Surgery, Royal Infirmary of Edinburgh, Edinburgh, United Kingdom

**Keywords:** subxiphoid, incisional, hernia, minimal invasive surgery, post sternotomy, abdominal wall reconstruction, epigastric hernia, cardiac surgery

## Abstract

**Introduction:**

Subxiphoid hernias are indeed an uncommon type of hernia that tend to present in the caudal aspect of a sternotomy incision, which typically enters the epigastrium. These patients have usually undergone major cardiac surgeries, like heart transplant, coronary artery bypass grafting (CABG), or cardiac valve replacement, representing a high-risk group of patients. The purpose of the study is to identify risk factors, prevention measures, and to explore different techniques for surgical management, including whether minimal invasive surgery is superior than the conventional open approach.

**Material and Methods:**

A comprehensive search was performed on Pubmed, Sciencedirect, Scopus, and Cochrane library. The search terms included “subxiphoid hernia” and “post sternotomy hernia.” Articles not in the English literature and duplicates studies were excluded. Studies regarding epigastric hernias were also excluded. All relevant articles published until 28th of February 2025 were included. Relevant references from the identified articles were also searched and included for review.

**Results:**

Particular care should be given to recognizing patient-related risk factors, preventing surgical site infections, and ensuring proper closure of the fascia. Regarding surgical management, seventeen articles were identified with 442 patients overall. 320 patients underwent open repair, while in 122 patients laparoscopic approach was achieved. Intraperitoneal onlay mesh placement was the most popular laparoscopic technique applied. Only 3 studies provided comparable results between the two approaches. A significant variety of techniques concerning both approaches was noticed in the literature.

**Conclusion:**

Excellent knowledge of the anatomical and physiological aspects of the subxiphoid region, and acknowledgement of risk factors, are essential. Minimal invasive repair of subxiphoid hernias is a feasible option, as long as defect closure and adequate mesh overlap are achieved. There are not enough data still to prove the superiority of the laparoscopic approach. Complex cases should be referred to experienced hernia surgeons.

## Introduction

Subxiphoid hernias (SH) typically present in the midline, usually within 3 cm from the xiphoid, and are classified as midline (M1) hernias according to the EHS (European Hernia Society) classification [1]. While they can also occur off midline, midline SH are more commonly observed [1]. The reported incidence is relatively low, ranging from 1% to 4.2% in patients who have undergone cardiac surgery [1–3]. However, the true incidence is likely underestimated due to the often asymptomatic nature of these hernias. Additionally, the anterior surface of the liver prevents intestinal incarceration within the hernia defect, which may further mask the condition’s clinical significance [4]. The lack of long-term follow-up for these patients also contributes to the underreporting of SH [5].

Kim et al [6] reported 0.8% of 1,656 cardiac bypass patients required SH repair, further emphasizing the low but notable occurrence of this condition in post-cardiac surgery patients. The basic principles of SH repair align with those for any abdominal wall defect, including tension-free repair, mesh placement beneath the fascia with ideally 5 cm of mesh overlap around all edges, and appropriate mesh fixation [5]. These principles are critical for ensuring a durable repair and minimizing recurrence. However, there are some unique challenges associated with repairing SH. Their proximity to the thoracic cage and the adherence of the heart to the scar, make their repair especially demanding and susceptible to failure [5]. The application of component separation in that area is particularly challenging due to anatomical restrictions, such as the attachment of the external oblique aponeurosis on the inferior ribs, and the fusion if the transversalis fascia with the parietal diaphragmatic peritoneum. As a result, recurrence rates remain high, with estimates ranging from 33% for mesh repair to 43% for sutured repair [7].

The purpose of this study is to reveal predisposing factors for the development of SH, identify prevention strategies, understand the specific anatomic considerations, and explore different surgical techniques, including both open and minimal invasive approaches.

## Methods

A comprehensive search was performed on Pubmed, Sciencedirect, Scopus, and Cochrane library. The search terms included “subxiphoid hernia” and “post sternotomy hernia.” A literature search was performed by two independent reviewers. Articles not in the English literature and duplicate studies were excluded. Studies regarding epigastric, ventral and diaphragmatic hernias, were excluded. Publications regarding subxiphoid hernias after laparoscopic cholecystectomy were also excluded. Only articles concerning subxiphoid hernias in adults following sternotomy, published from January 1985 until 28th of February 2025, were included. Relevant references from the identified articles were also searched and included for review.

## Results

### Predisposing Factors and Prevention

SH can develop following various types of surgical procedures including median sternotomy, midline epigastric laparotomy, or chevron incisions [8]. These procedures are often employed for cardiac or upper abdominal surgeries, making SH a notable complication in these contexts.

Several patient-related risk factors are frequently associated with the development of SH. These include obesity, male sex, advanced age, surgical site infections (both superficial and deep), left-sided heart failure, low cardiac output syndrome, long incisions, reoperations, heart transplant surgery, immunosuppression, as well as conditions like diabetes mellitus (DM), smoking, chronic obstructive pulmonary disease (COPD), history of other hernias, and postoperative bleeding that requires early transfusion [1, 3, 6, 9–11]. Notably, only one single-center study by Kim et al [6] has identified female gender as a risk factor for SH formation, which contrasts with the general male predominance found in the majority of the literature. Weight loss for obese patients and avoidance of reoperation when possible may be considered [12].

Technical factors also play a crucial role in SH development. Inadequate incision techniques, improper closure methods, and the use of absorbable sutures, are associated with an increased likelihood of hernia formation. These technical issues can contribute to weak abdominal wall closure, leading to the formation of hernias over time.

Barner [13] proposed a modification of median sternotomy to reduce the occurrence of SH. This modified approach involved using a shorter incision that stopped before the xiphoid process and angled off midline, towards the left xiphoid-costal angle. This technique provided adequate exposure for the procedure while avoiding disruption of the linea alba, which is a key structure in abdominal wall integrity. Notably, none of the 2,500 patients who underwent surgery with this technique developed SH. However, it is important to exercise caution during these approach to avoid injury to the left superior epigastric artery, which supplies vital blood flow to the abdominal wall.

Davidson [3, 12, 14] further suggested using non-absorbable sutures to close the linea alba. This technique ensures a more durable abdominal wall closure, potentially reducing the risk of hernia development post-surgery.

Wound infection and immunosuppression have been recognized as significant predisposing factors for SH recurrence after surgical repair [1, 9]. Effective and early diagnosis and management of wound infections are critical to preventing both the development of SH and its recurrence [9].

### Clinical Presentation and Diagnosis

Most SH are small and asymptomatic and as a result they remain undiagnosed, as only symptomatic patients seek medical attention, indicating that the real incidence is underestimated [5]. Specifically, the percentage of symptomatic SH varies widely, ranging from 35% to 100% [3, 5]. These hernias usually develop within the first 3–4 years postoperatively [8]. Epigastric pain, bulging, nausea, vomiting are some of the symptoms reported [14, 15]. It is speculated that the underlying liver prevents bowel incarceration within the hernia defect [15, 16]. Patients with symptomatic SH are generally treated electively [2]. However, urgent surgical intervention due to incarceration has been also reported in the literature [2]. Liver incarceration has also been described [17]. These cases highlight the potential for SH to cause significant complications if left untreated or undiagnosed for too long.

Physical examination in combination with ultrasound or computed tomography (CT) are necessary for the confirmation of the diagnosis [15]. Computed tomography (CT) of the chest and abdomen is essential for assessing the extent of the hernia [8]. Dynamic CT with Valsalva manoeuvre may also be useful [11]. CT imaging can provide detailed information about the hernia’s size, which can vary significantly, with some hernias reaching up to 16 cm in the longitudinal axis [18], while the average size typically ranges from 2 to 15 cm [8]. This imaging helps to plan the appropriate surgical approach and anticipate any challenges that might arise during repair.

Preoperative assessment should include a comprehensive cardiac workup, as many of the patients suffer from an underlying cardiac condition [8]. This evaluation ensures that the surgical team is fully informed about the patient’s cardiac status, which is crucial for optimizing the perioperative management and minimizing potential complications.

### Surgical Management

Overall, seventeen articles were identified with 442 patients in total. 320 patients underwent open repair, while in 122 patients laparoscopic approach was achieved. The majority of the studies are single center retrospective studies. Only one multicenter study with a larger sample was identified. Two studies were prospective, while only three studies provided comparative outcomes between open and laparoscopic approach. Case reports are also included. A brief summary of the literature is illustrated in Table 1.

**TABLE 1:** Brief summary of the literature.

Study, year	Number of patients	Type of study	Type of procedure	Special considerations	Mesh	Fixation	Follow up (months)	Complications	Recurrence
Cohen and starling [14], 1985	14	Single center retrospective study	Open	Excision of bifid xiphoid process	Marlex	Fascial sandwich anchored To the musculofascial edge	4–36	None	0
Davidson and bailey [12], 1987	8	Single center retrospective study	Open Primary repair	5 direct closure, 3 modification of Well’s procedure	NA	NA	8–43	NA	0
Bouillot et al [19], 1997	23	Single center retrospective study	Open	Rectorectus	Dacron mesh	If the closure is under tension, the rectus Sheath can be relaxed by multiple staggered Overlapping 8 and lo mm incisions	12–60	3 hematomas	0
Landau at al [2], 2001	10	Single center retrospective study	Laparoscopic	Intraperitoneal onlay	Gore-Tex	2–0 vicryl sutures, tacks	10–42	3/10 (2 minor, 1 small bowel obstruction treated with laparoscopy)	1
Mackey et al [9], 2005	45	Single center retrospective study	14 primary repair, 21 open repair with mesh, and 10 laparoscopic repair with mesh	NA	31 mesh, 14 primary repair	NA	NA	1 sternal wound infection	Overall 36%, 43% recurrence after primary repair, 10 (32%) recurrences in the mesh repair group (7 open, 3 laparoscopic)
Eisenberg et al [20], 2008	4	Single center retrospective study	Laparoscopic	Intraperitoneal onlay	Gore dual mesh in 2 patients, parietex mesh was in 2	Transfascial nonabsorbable sutures and spiral tacks	NA	1 ileus, 1 pulmonary oedema	NA
Ferrari et al [21], 2009	15 (2 after median sternotomy)	Single center retrospective study	Laparoscopic	Intraperitoneal onlay	Gore dual	Intraperitoneal stitches and double crown tacks	Mean 37	Non specified	6.6%
Tatay et al [11], 2011	35 (10 after sternotomy)	Prospective single center study	Open	Double mesh technique (preperitoneal and supra-aponeurotic)	Polypropylene mesh	Monofilament sutures, fibrin	NA	1 wound infection, 9 seromas	0
Kim et al [6], 2012	13	Single center retrospective study	Open	NA	Marlex or prolene	NA	NA	NA	0
Shah et al [4], 2013	1	Case report	Single incision laparoscopic	Intra peritoneal onlay Dual layered meshplasty	NA	Four transfascial sutures and absorbable tacks	NA	NA	NA
Vennarecci et al [16], 2015	1	Case report	Open	Sublay	Permacol	NA	6 m	0	0
Ghanem et al [8], 2016	4	Prospective single center study	Laparoscopic	Incision of posterior rectus sheath to achieve tension free suturing, or intraabdominal onlay bridging mesh for defects >7–10 cm	NA	Nonabsorbable intracorporeal sutures to anchor the mesh to the diaphragm above the costal margins. Transfascial nonabsorbable sutures and tacks below the costal margin.	12 m	0	0
De mesquita et al [18], 2017	15	Retrospective single center study	Open	Vertical relaxing incision	Marlex, onlay	Absorbable sutures	7–33 months	2 hematomas, 3 partial wound dehiscence	0
Raakow et al [15], 2018	28	Single center retrospective study	20 open, 8 laparoscopic	Hernia Defect not closed in the laparoscopic approach	Ultrapro mesh (14), vypro (4), optilene (2), sublay, composite mesh for laparoscopic (IPOM)	Intraabdominally anchoring sutures, absorbable tacks, fibrin glue	Median after open repair 48.8 (8–76 months), 32.5 (4–68 months) after laparoscopic	Severe complications 3/20 for the open group which required reoperation, no severe complications or the laparoscopic group	No recurrences in the open group, 3 recurrences in the laparoscopic (*p* = 0.031)
Albrecht et al [10], 2020	208	Multicenter quality assurance study	Open 139, and laparoscopic 69	92 open sublay, 22 open IPOM, 10 open onlay, IPOM for all laparoscopic procedures	Non-absorbable	Sutures and/or endoscopic tacks	12	No significant differences between the groups	Laparoscopic group 7.2 vs. open 2.2%; p = 0.072
Misumi et tal [17], 2021	1	Case report	Laparoscopic	Intraperitoneal onlay	Ventrio	Transfascial sutures	18 months	0	0
Abello et al [22], 2021	42	Retrospective single center study	Open	22 preperitoneal, 20 adjusted double mesh	ΝΑ	ΝΑ	Average 25.8 ± 15.1	Minor complications grade I (according to clavien dindo classification)	No statistically significant differences in hernia recurrence (*P* = 0.288)

NA, not applicable.

Recurrence rates following SH repair have been reported to range from 24% to 44% [6], highlighting the challenging nature of the procedure. The high rates of recurrence following primary suture repairs, which can range from 43% to 80% [5, 9], underscored the need for improved techniques and materials. This led to the adoption of polypropylene mesh for open surgical repair, a practice first introduced by Cohen and Starling in the 1980s. They used a preperitoneal approach, entering the peritoneal cavity only when necessary to free adhesions, which helped minimize surgical trauma [14]. Davidson and Bailey [12] described the application of a double door flap as a modified Well’s procedure, for the repair of large subxiphoid hernias more than 10 cm, with zero recurrence rates after three and a half years of follow up, however this technique was applied in only three patients. The introduction of a permanent mesh significantly reduced the recurrence rates. Studies have reported recurrence rates between 0% and 32% wish mesh repair [5, 9], demonstrating a clear improvement compared to suture-only methods.

There are several approaches for mesh placement in SH repair. The main techniques include onlay [10, 18], sublay [10, 15, 16], preperitoneal [22], rectorectus [19], and intraperitoneal onlay mesh (IPOM) placements. A variety of mesh placement approaches is noticed even within the same studies, while not all of them provide sufficient information. In addition, different kind of meshes and sutures were used, which makes it difficult to compare and reach safe conclusions. Τhe onlay approach is the simplest and the most reproducible technique, but it is associated with higher rates of recurrence, seroma formation, and infection [5]. The rectorectus approach usually allows adequate mesh overlap and is considered a reliable approach for achieving a stable repair [23]. Intraperitoneal onlay mesh placement was applied in all the laparoscopic procedures, as shown in Table 1. In addition, Awad et al [7] adjusted the Rives-Stoppa-Wantz repair to the SH repair by detaching the posterior sheath from its insertion to the posterior aspect of the xiphoid, and placing the subxiphoid part of the mesh intraperitoneal. Care should be given to avoid encircling the ribs with the mesh, as the costal perichondrium is very well innervated, and such placement can lead to persistent pain postoperatively [5].

Although the closure of the SH defect is important, large defects or increased tension make it particularly challenging. The Clotteau method involves multiple incisions in the external oblique aponeurosis to allow relaxation and approximation of the linea alba in the midline in combination with mesh placement [5, 18]. Multiple vertical relaxing incisions on the anterior rectus sheath were applied by Bouillot et al [19] and de Mesquita et al [18], in combination with rectorectus and onlay mesh placement respectively, with no recurrences reported in the follow up. When laparoscopic approach is used, the hernia defect is usually not sutured. Ghanem et al [8] applied incisions in the posterior rectus sheath laparoscopically, for the closure of the abdominal wall defect when possible. Otherwise, intraperitoneal onlay bridging was preferred for larger defects [8].

Another method suggested in order to overcome the tension related to the hernia wall closure, is the application of two meshes. Tatay et al [11] described a double mesh technique, with one mesh applied preperitoneal and one supra-aponeurotic, to avoid any tension. This technique was performed in 35 patients, 10 of which following heart procedure, with no recurrence rates. Abello et al [22] applied the same adjusted double mesh technique in 20 patients and the outcomes were compared to the conventional preperitoneal mesh placement performed in 22 patients in terms of an observational non randomized study. No statistically significant differences in hernia recurrence was found between the two groups (*P* = 0.288), suggesting there is not enough evidence to support the double mesh technique.

In 2001, Landau et al [2] described the first laparoscopic repair of post-sternotomy subxiphoid epigastric hernia. The laparoscopic approach allows better visualization of the hernia defect, minimizes tissue trauma, avoids previously infected sternal wounds, and reduces operative time [9, 15]. For a laparoscopic repair, adhesiolysis and takedown of the falciform ligament up to the hepatic veins are mandatory steps to fully expose the hernia defect and ensure that there is adequate mesh overlap (approximately 5 cm) [5, 8, 10, 23]. A wider overlap of 7–10 cm laterally for larger defects has been suggested by Ghanem et al [8]. In case no sutures are placed above the costal margin, an additional overlap of 8 cm superiorly has been proposed, to make sure that the liver holds the mesh during desufflation of the peritoneal cavity [24].

In the repair of ventral hernias, proper mesh fixation with sutures and tacks is crucial to prevent recurrence and complications, due to possible dislocation of the mesh [17, 20]. However, in terms of a laparoscopic subxiphoid hernia repair, neither sutures nor tacks are placed in the cephalad part, above the costal margin [20]. When tacks are used, they should be placed at or below the costal margin, and definitely not above it, as they might cause chronic pain or pericardial injury resulting in complications such as pericarditis and cardiac tamponade [23, 25]. In fact, the mortality rate associated to pericardial or heart muscle injury after tack fixation can be as high as 48%, despite surgical intervention [24]. Instead, nonabsorbable intracorporeal sutures can be applied superficially to fix the mesh to the diaphragm and encourage scarring, with caution after grasping the diaphragm [8, 17, 18]. Some surgeons prefer not to fix the mesh above the costal margin at all, relying on the liver and stomach to secure the mesh in place [20]. The use of fibrin glue for the fixation of the mesh on the cranial side has been explored, but it has been associated with high recurrence rates [15, 24].

Mesh fixation using only tacks is not recommended, as they penetrate the mesh for only 2 mm [5]. Therefore, additional full-thickness sutures placed circumferentially every 3–6 cm have been suggested to ensure fixation especially when fascial closure is not achieved, always below the costal margin [5, 8]. For laparoscopic repair, a fascial closure device, such as Endoclose or Endoclinch, may be helpful [2, 8]. Hope and Hooks [23] suggested placing the most superior stitch right below the xiphoid or on the lateral side of the xiphoid. Finally, fixing the mesh laterally to the rectus muscles offers better mechanical stability and reduces the risk of injury to the epigastric vessels, which could result in a hematoma or require reoperation [26].

When intraperitoneal mesh is used, a dual-surface material is preferred, to avoid potential complications, such as adhesions and bowel fistula [2]. In this review, dual-surface mesh was used in all IPOMs, when relevant information was provided [2, 4, 10, 17, 20]. Regarding postoperative complications concerning abdominal viscera, only one small bowel obstruction requiring laparoscopical repair in the early postoperative period, was reported [2], while one patient developed ileus managed conservatively [20]. For open repair, non-absorbable meshes usually from polypropylene [10, 11, 14, 18] or polyester [19] are preferred. Only one study mentioned the application of partially absorbable meshes with no recurrence rates [15], while only one case with biological mesh placement in a heart transplant recipient is reported in the literature with a follow up period of 6 months [16].

Complications such as hematomas [18, 19], seromas [11], wound infection [9, 11], partial wound dehiscence [18], or other severe complications requiring reoperation [15], appear to be more frequent in the open repair, as seen in Table 1. However, no statistical significant difference was found in the study published by Albrecht et al [10], where the sample size was larger.

The recurrence rates may be further reduced with laparoscopic repair, with reported rates as low as 10% [2, 18], however there are not enough data to support the superiority of the laparoscopic approach. Only 3 comparative studies were identified in the literature. Mackey et al [9] reported 30% (3/10) recurrence in the laparoscopic and 33.3% (7/21) recurrence in the open group. Raakow et al. [15] reported higher recurrence rates in the laparoscopic group (p = 0, 031). However, the sample size was small, including 20 patients in the open, and 8 patients in the laparoscopic group, while the open group was associated with higher rates of lost follow-ups. In addition, the hernia defect was not primarily closed in the laparoscopic group. For these reasons, the increased recurrence rates after laparoscopic surgery as reported in this study should not be considered discouraging. On the other hand, Albrecht et al [10] in a multicenter study with 208 participants in total, found no statistical significant difference regarding recurrence rates (laparoscopic group 7.2 vs. open 2.2%; p = 0.072) after 1 year of follow up.

The use of an abdominal binder for the first 4–6 postoperative weeks has been proposed by Raakow et al. [15], beginning right after the end of the procedure. The role of drains in seroma prevention remains unknown [15].

Special consideration should be made in heart transplant recipients undergoing SH repair. These patients often require the expertise of a dedicated cardiac anesthesiologist to manage their complex cardiac status. Additionally, lower intraabdominal insufflation pressures are recommended, and continuous monitoring of cardiac function is essential [20]. A combination of general and epidural anesthesia is also suggested to enhance pain control and promote faster recovery [4]. For high-risk patients undergoing open repair, bilateral ultrasound-guided transverse abdominis plane (TAP) block has been proposed as an effective method for providing regional anesthesia. This technique avoids intubation and hemodynamic alterations during surgery [27]. It also prevents postoperative pulmonary complications, nausea, and achieves better analgesia, leading to earlier mobilization [27].

## Discussion

Repairing SH is a particularly challenging procedure due to several anatomical and physiological factors that complicate the surgical approach. One of the primary challenges is the increased lateral tension created by structures such as the costoxiphoid ligament, the transversus thoracis, and the sternal portion of the diaphragm. These structures make it difficult to approximate the borders of the rectus abdominalis sheath under minimal tension, which is essential for a stable repair [13]. Respiration and coughing contribute further to the lateral tension, and the increased intraabdominal pressure [2, 18].

From an anatomical standpoint, the rectus muscles and the anterior rectus sheath attach to the xiphoid process anteriorly, while the posterior rectus sheath and the diaphragm attach to the it posteriorly [5]. Perixiphoid and subxiphoid fat lies between the xiphoid process and the diaphragm. In addition, the close proximity to the ribs, diaphragm, and central tendon, leaves limited space for the fixation of a mesh, especially given the narrow retro-xiphoid space [1]. Another significant concern is the potential for anatomic variations such as a bifid or divided xiphoid. Furthermore, the blood supply to the xiphoid process is achieved through the ensiform vessels, which typically derive from the internal thoracic artery, as terminal branches, or alternatively the superior epigastric artery, and it may be compromised [1]. A disrupted blood supply can further complicate the procedure and affect the healing process.

Obesity, male sex, wound infections, low cardiac output, long incisions, reoperations, heart transplant, immunosuppression, DM, smoking, COPD, need for transfusion [1, 3, 6, 9–11] suggest risk factors for the development of SH. Avoiding the disruption of the linea alba in the midline [13], application of non-absorbable sutures for closure [3], preoperative weight loss [12], early recognition and management of wound infections, may decrease the risk of SH development. Epigastric pain, bulging, nausea, vomiting are some of the symptoms reported [14, 15] when patients seek medical advice. A variety of techniques regarding mesh placement for SH repair have been described, such as onlay [10, 18], sublay [10, 15, 16], preperitoneal [22], rectorectus [19], and IPOM, with IPOM being the procedure of choice for laparoscopic approach. There is not enough evidence supporting the superiority of the laparoscopic approach so far.

According to a recent Delphi consensus [28], both open and minimally invasive procedures are considered appropriate for SH repair, provided that defect closure and adequate mesh overlap are achieved. The key to a successful repair lies in comprehensive understanding of the complex anatomy of the area. This includes detaching the posterior rectus sheath and placing the mesh in the extraperitoneal space, which are crucial steps to prevent complications and ensure a robust repair [28]. For larger hernia defects (greater than 4 cm) or cases where closure is difficult, a rectorectus repair combined with transversus abdominis release (TAR) performed by experienced hernia surgeons may be an appropriate solution [28]. Preoperative botulinum toxin A (BTA) administration has also been explored in combination with external oblique release to improve outcomes. However, its use in this context remains controversial, as it did not demonstrate significant benefits [29]. A cost-effective silicone model, mimicking human tissue, is available for training, specifically for open retro-muscular mesh implantation and the preparation of the fatty triangle, which can be challenging especially for beginners [30].

There is currently insufficient evidence to support the superiority of either the open or laparoscopic approach for SH repair. A few studies have examined both methods, but variation in study design and sample size complicate direct comparisons. Raakow et al [15] noticed significantly higher recurrence rates in the laparoscopic group compared to the open approach. However, the sample size of the laparoscopic group was much smaller, and it is possible that the learning curve for laparoscopic repair had not yet been fully overcome. Similarly, Albrecht et al [10] in a retrospective multicenter study also found higher recurrence rates in the laparoscopic group after 1 year, although the difference did not reach statistical significance. It is worth noting that fascial closure was not performed in all cases within the laparoscopic group, which raises questions about the role of this key step in recurrence rates. However, a steep learning curve is required for optimal results [1], and given the rarity of SH, it is difficult to achieve this proficiency consistently.

In terms of advancements, robotic-assisted repair of SH with suprapubic approach has also been proposed, offering the advantage of a better intraoperative view in comparison with the lateral approach [31]. The robotic approach may enable easier closure of the hernia defect, which appears to be difficult with the conventional laparoscopic approach, although training is required. Additionally, single-incision laparoscopic repair of SH has been described as a promising option, providing a better aesthetic outcome and potentially less postoperative pain [4].

There are certain limitations in regards with this study. Most of the literature is based on single-center retrospective studies with small number of patients. A variety of surgical techniques and materials are described, even within the same study. The follow up period is short, less than 4 years. As this study is not a systematic review, it intrinsically contains a subjective part. The asymptomatic character and the rare incidence of SH does not allow randomized control trials. An international multi-center registry with long follow-up would be helpful for data collection and further analysis.

## Conclusion

While SH are rare, they pose significant surgical challenges. The development of SH is multifactorial with a combination of patient characteristics, surgical history, and technical factors contributing to their formation. Meticulous attention to repair technique and consideration of anatomical complexities are essential to improving outcomes and reducing recurrence rates. Both open and minimally invasive procedures are considered appropriate. The learning curve and technique-specific factors such as fascial closure and mesh fixation may impact long-term success.
